# Ammonium Chloride Associated Aerosol Liquid Water Enhances Haze in Delhi, India

**DOI:** 10.1021/acs.est.2c00650

**Published:** 2022-04-28

**Authors:** Ying Chen, Yu Wang, Athanasios Nenes, Oliver Wild, Shaojie Song, Dawei Hu, Dantong Liu, Jianjun He, Lea Hildebrandt Ruiz, Joshua S. Apte, Sachin S. Gunthe, Pengfei Liu

**Affiliations:** † Lancaster Environment Centre, Lancaster University, Lancaster LA1 4YQ, U.K.; ‡ College of Engineering, Mathematics and Physical Sciences, University of Exeter, Exeter EX4 4QE, U.K.; § Laboratory of Atmospheric Chemistry, Paul Scherrer Institut (PSI), Villigen 5232, Switzerland; ∥ Institute for Atmospheric and Climate Science, ETH Zurich, Zurich 8006, Switzerland; ⊥ School of Architecture, Civil & Environmental Engineering, École Polytechnique Fédérale de Lausanne, Lausanne 1015, Switzerland; # Center for the Studies of Air Quality and Climate Change, Institute of Chemical Engineering Sciences, Foundation for Research and Technology Hellas, Patras 26504, Greece; ∇ John A. Paulson School of Engineering and Applied Sciences, Harvard University, Cambridge, Massachusetts 02134, United States; ○ College of Environmental Science and Engineering, Nankai University, Tianjin 300071, China; ● Centre for Atmospheric Sciences, Department of Earth, Atmospheric and Environmental Sciences, University of Manchester, Manchester M13 9PS, U.K.; ◆ Department of Atmospheric Sciences, School of Earth Sciences, Zhejiang University, Hangzhou, Zhejiang 310058, China; ▲ State Key Laboratory of Severe Weather & Key Laboratory of Atmospheric Chemistry of CMA, Chinese Academy of Meteorological Sciences, Beijing 100081, China; △ McKetta Department of Chemical Engineering, The University of Texas at Austin, Austin, Texas 78712, United States; ¶ Department of Civil and Environmental Engineering, UC Berkeley, Berkeley, California 94720, United States; ☆ EWRE Division, Department of Civil Engineering, Indian Institute of Technology Madras, Chennai 600036, India; ★ Laboratory for Atmospheric and Climate Sciences, Indian Institute of Technology Madras, Chennai 600036, India; ▼ School of Earth and Atmospheric Sciences, Georgia Institute of Technology, Atlanta, Georgia 30318, United States

**Keywords:** Air pollution, Secondary inorganic aerosol, Hygroscopicity, Particulate matter, Heterogeneous formation

## Abstract

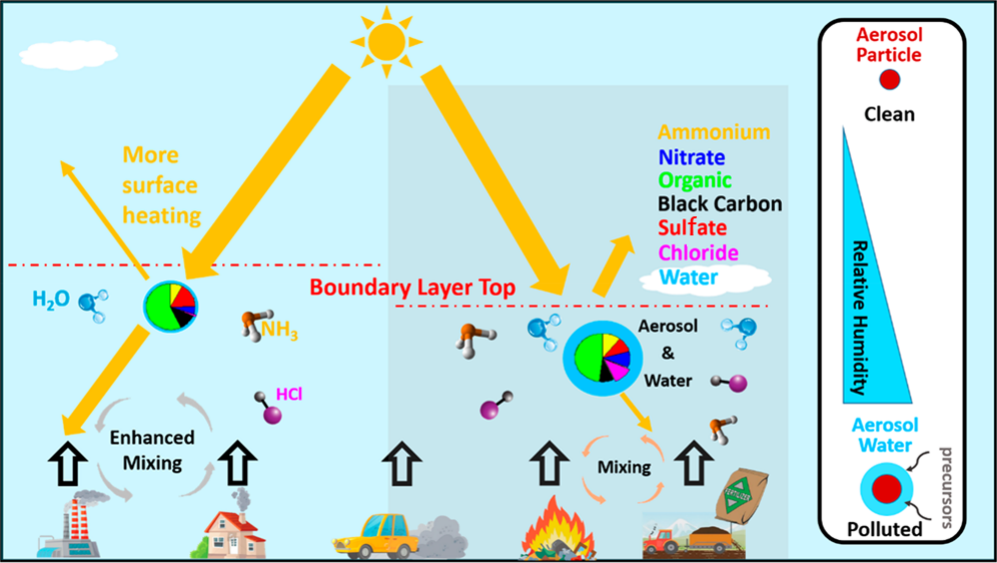

The interaction between water vapor and atmospheric aerosol leads to enhancement in aerosol water content, which facilitates haze development, but its concentrations, sources, and impacts remain largely unknown in polluted urban environments. Here, we show that the Indian capital, Delhi, which tops the list of polluted capital cities, also experiences the highest aerosol water yet reported worldwide. This high aerosol water promotes secondary formation of aerosols and worsens air pollution. We report that severe pollution events are commonly associated with high aerosol water which enhances light scattering and reduces visibility by 70%. Strong light scattering also suppresses the boundary layer height on winter mornings in Delhi, inhibiting dispersal of pollutants and further exacerbating morning pollution peaks. We provide evidence that ammonium chloride is the largest contributor to aerosol water in Delhi, making up 40% on average, and we highlight that regulation of chlorine-containing precursors should be considered in mitigation strategies.

## Abstract 2

This research highlights the role of ammonium chloride in enhancing air pollution in Delhi through aerosol water content, allowing us to suggest suitable mitigation strategies.

## Introduction

Particulate matter (PM) pollution is a major threat to the atmospheric environment around the world, impacting public health, visibility, ecosystems, climate, and economics.^1−10^ Aerosol liquid water content (ALWC), namely the condensed water associated with aerosol particles, represents a substantial fraction of the mass of tropospheric particulate matter.^11,12^ ALWC can exacerbate PM pollution; however, its concentration, sources, and impacts are largely unknown in heavily polluted urban environments such as that in Delhi.

Aerosol water not only contributes significantly to total aerosol mass but also greatly influences uptake of gaseous precursors^3,13−15^ and enhances light scattering,^16−19^ which affect secondary formation of PM and photochemistry in the atmosphere.^20,21^ Furthermore, ALWC can reduce the consistency of aerosol observations by increasing the size of particles and changing their collection efficiency, therefore hampering robust analysis of formation mechanisms and spatiotemporal variations unless corrections are applied.^22^ ALWC is dependent on ambient relative humidity (RH), temperature, particulate matter mass concentration, aerosol phase state, and chemical composition.^23,24^ Thermodynamic models are often used to estimate the ALWC, because direct measurements remain challenging. A previous study, in which Delhi was not investigated, reported that Beijing experienced the highest ALWC loading among all sites studied globally.^25^ The ALWC contribution can double particulate matter loading in Beijing, with daily averages of up to 210 μg/m^3^.^26,27^ The high concentration of secondary inorganic aerosol in winter in Beijing, in particular following the co-condensation of nitrate with water vapor,^3,45^ was found to be the driving factor for ALWC production that facilitated haze development.^26^ Particulate matter pollution in the Indian capital Delhi is comparable or even more severe than that in Beijing^28^ and leads to ∼10,000 premature deaths per year in Delhi^29−34^ In situ observations indicate that aerosol in Delhi has a greater capacity to take up water than in Beijing,^19,34^ and ALWC may therefore play a more critical role in the deterioration of air quality. Our previous work, based on a one-month winter-case study, demonstrates that high hygroscopicity of aerosol in Delhi is largely due to the co-condensation of ammonium chloride, which greatly enhances visibility reduction and cloud condensation nuclei activation in winter.^33^ However, a thorough understanding of the local characteristics in different seasons, quantifying the contribution of ammonium chloride to ALWC and providing deeper insight into the impact of ALWC on atmospheric physiochemical processes in Delhi, is still lacking. This gap of current knowledge hampers development of more effective and targeted mitigation strategies to improve air quality in Delhi.

To address this gap, we investigate and characterize ALWC in Delhi using long-term observations of the composition of submicron aerosol particles (referred to here as PM_1_),^32,35^ and we further derive the contributions to ALWC from each aerosol component to better understand its source using a thermodynamic model.^24^ We report that ALWC in Delhi is much higher than in any other locations previously reported^25^ and quantify the average contribution of ammonium chloride to total ALWC. We further perform a comprehensive analysis to demonstrate how this uniquely high ALWC influences the development of the planetary boundary layer (PBL) and the secondary formation of PM in Delhi. Our results shed light on the development of haze pollution in Delhi and permit formulation of better targeted mitigation strategies for the city.

## Materials and Methods

### Observations

Comprehensive, near-continuous in situ observations of ambient aerosol particles have been made at the Delhi Aerosol Supersite at the Indian Institute of Technology Delhi campus in South Delhi (77.191° E; 28.546° N) since January 2017.^32,36−38^ The observations at this supersite represent the overall pollution conditions in Delhi well, and a recent observational study across multiple sites has shown that the sources and characteristics of particulate matter are relatively homogeneous across the city.^39^ For the present analysis, we employ previously published observations from January 2017 to March 2018, which encompass two winter periods.^32,35,37^ An Aerosol Chemical Speciation Monitor (ACSM; Aerodyne Research, Billerica, MA) was employed to observe the nonrefractory chemical components (including nitrate, sulfate chloride, ammonium, and organics) in PM_1_, and black carbon was measured simultaneously using a multichannel aethalometer (Magee Scientific Model AE33, Berkeley, CA). Most of the observed particulate chloride is likely to be present as ammonium chloride, because the chloride mass fraction has a strong positive correlation with the ammonium fraction and is negatively correlated with the organic fraction (Figure S1), while the other forms of chloride such as the refractory potassium and sodium chloride are not observed by ACSM. In addition, the ion balance of sulfate, nitrate, and chloride against ammonium is close to 1:1, but the abundance of anions is otherwise about 45% less than that of cations if chloride is excluded (Figure S2). This is consistent with ref (33), which found using independent measurements that most of the chloride in winter haze events in Delhi was present as ammonium chloride. Fine particle number size distribution (PNSD) was also monitored using a scanning mobility particle sizer (SMPS; TSI, Minnesota, USA). The instruments were well calibrated and operated in a temperature-controlled laboratory at the site. Detailed correction, calibration, and operational procedures are given in ref (32). Hourly observations are available from ref (35) for the aerosol composition data set and from refs (37 and 40) for the PNSD data set, which are adopted to estimate ALWC and the rate constant of heterogeneous loss of SO_2_ and N_2_O_5_ in this study.

Hourly surface meteorological conditions, including RH, temperature, visibility, wind speed, and direction, at the Indira Gandhi International Airport which is 8 km from the Supersite, are taken from the National Oceanic and Atmospheric Administration Integrated Surface Database (https://www.ncdc.noaa.gov/). Hourly downward solar radiation at the surface and the height of the planetary boundary layer are obtained from the European Center for Medium-Range Weather Forecasts reanalysis data set (ERA5, https://www.ecmwf.int/) at 0.25° × 0.25° spatial resolution. Hourly surface concentrations of SO_2_ and NO_2_ at P K Puram (77.187^o^ E; 28.563^o^ N), which is only 5 km from the Supersite, are taken from the Indian Central Pollution Control Board database (http://www.cpcb.gov.in/). The observations at the P K Puram site are operated by the Delhi Pollution Control Committee and are well calibrated and quality controlled with reported error typically under 5%.^41^Figure S3 shows an overview of all aforementioned observational data sets.

In order to estimate the influence of ALWC-enhanced light extinction on surface radiation and the development of the planetary boundary layer (PBL), solar radiative transfer calculations were performed using the Tropospheric Ultraviolet and Visible Radiation model (TUV, v5.3.2) developed at NCAR (https://www2.acom.ucar.edu/modeling/tuv-download). During winter and spring in Delhi, the average ozone column loading is ∼270 DU, the single scattering albedo of aerosol observed by Aura-OMI is ∼0.8, surface albedo provided by MERRA-2 analysis is 0.2, and the average aerosol optical depth of ambient wet aerosol particles observed by Terra-MODIS is 0.74 in winter and 0.53 in spring (Collection 6.1, Level-3 monthly data set).

### Aerosol Liquid Water Content

The ALWC associated with inorganic components is calculated from the meteorological variables and ACSM observations of aerosol chemical composition using the thermodynamic model ISORROPIA (version 2.1),^24^ assuming a metastable state for aerosol particles without solid precipitates. This assumption is reasonable because only less than 5% of the period is under a condition of RH < 20% where aerosol particles are unlikely presented as liquid state^24,42,43^ and contribute negligible ALWC in our study. Following previous work,^44^ we further develop the model to estimate the relative contributions of different electrolytes to water uptake. Due to a lack of gaseous observations, the reverse mode of ISORROPIA, which only requires particle-phase chemical composition as input, is chosen for this study. A previous study has shown that there is little difference in ALWC produced using the forward and reverse modes, with a slope of 0.996 and an *R*^2^ of 0.988.^26^ The output of ISORROPIA is provided in the supplementary data set, with detailed guidance in its user manual (http://isorropia.epfl.ch), and species-wise ALWC is added. The ISORROPIA model was validated theoretically against the benchmark thermodynamic model E-AIM.^45,46^ The ISORROPIA model has been widely used to estimate ALWC worldwide^25,26,33,47^ and has been well validated against that derived from visibility reduction in Delhi^19,33^ and from direct observations of aerosol hygroscopicity in Beijing.^26,47^ Many global and regional atmospheric chemistry transport models, e.g., GEOS-Chem,^48^ CMAQ,^49^ and NAQPMS,^50^ employ ISORROPIA to perform thermodynamic calculation and are well validated worldwide. The ALWC associated with organic compounds is estimated using the κ-Köhler theory,^23,51^ where a κ value of 0.1 is adopted for bulk organics in accordance with recent works in Delhi,^33,34^ and black carbon is assumed to be hydrophobic (i.e., κ = 0).

The κ of dry PM_1_ is derived from aerosol chemical composition using the Zdanovskii-Stokes-Robinson mixing method, following previous work.^22,51,52^ The light extinction enhancement factor due to ALWC, *f*(RH), can then be estimated from κ, RH, and the reference RH under dry conditions using eq 2 in ref (19). Here, the reference RH is taken as the average RH below 30%. This physically based empirical approach is well validated in Delhi.^33^ The relative contributions of dry PM_1_ and ALWC to visibility impairment is estimated using the equations below.
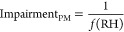
1
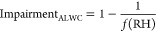
2The estimated ALWC is categorized by seasons to analyze the seasonal variation. According to the Indian National Science Academy (https://www.insaindia.res.in/climate.php), the climate of Delhi is conventionally characterized by five seasons: winter (December to January), spring (February to March), summer (April to June), the monsoon (July to September), and autumn (October to November). However, due to the lack of ACSM data in autumn, we exclude autumn from our analysis.

### Heterogeneous Loss of Trace Gases

To demonstrate the influence of ALWC on secondary formations of nitrate and sulfate via enhancement of heterogeneous reactions, we estimate the reaction rate constants (*k*) for heterogeneous loss of their precursors for wet and dry particles. Following previous work, we demonstrate this using N_2_O_5_ hydrolysis as a typical example of a heterogeneous pathway for nitrate formation^3,13,53^ and SO_2_ heterogeneous oxidation as an example for sulfate formation.^54^ Two four-day periods, a polluted period (12th–16th January 2018) and a relatively clean period (25th–29th April 2017), are selected for analysis to show how ALWC can enhance sulfate and nitrate formation in Delhi. These periods are selected because of the striking contrast in pollution levels and because both periods have all the required observations for estimating heterogeneous reaction rate constants. The reaction rate constants are estimated according to ref (55), as shown in eq 3
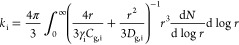
3where *k*_i_ is the reaction rate constant for trace gas i, representing N_2_O_5_ or SO_2_ in this study; γ_i_ is the uptake coefficient of gas i; *C*_g,i_ is the kinetic velocity of the molecules of gas i, which is calculated using eq 4 (in ref (3)); *D*_g,i_ is the diffusion coefficient of gas i, 0.85 × 10^–5^ m^2^/s for N_2_O_5_^56^ and 1.32 × 10^–5^ m^2^/s for SO_2_;^57^*r* is the radius of the particles, where the ALWC-derived growth factor is used to calculate *r* for wet particles; and “d *N*/d log *r*” is the particle number size distribution, with *N* representing the particle number concentration. Here, we estimate the γ_N_2_O_5__ following the method of ref (58), which accounts for the influences of RH, temperature, particle composition, and secondary organic coating (approximately 60% of total organics based on observations in Delhi^32^). We estimate the γ_SO_2__ as a function of RH, following the method of ref (54).

## Results and Discussion

### Seasonal and Diurnal Variations of ALWC

**Figure 1:**
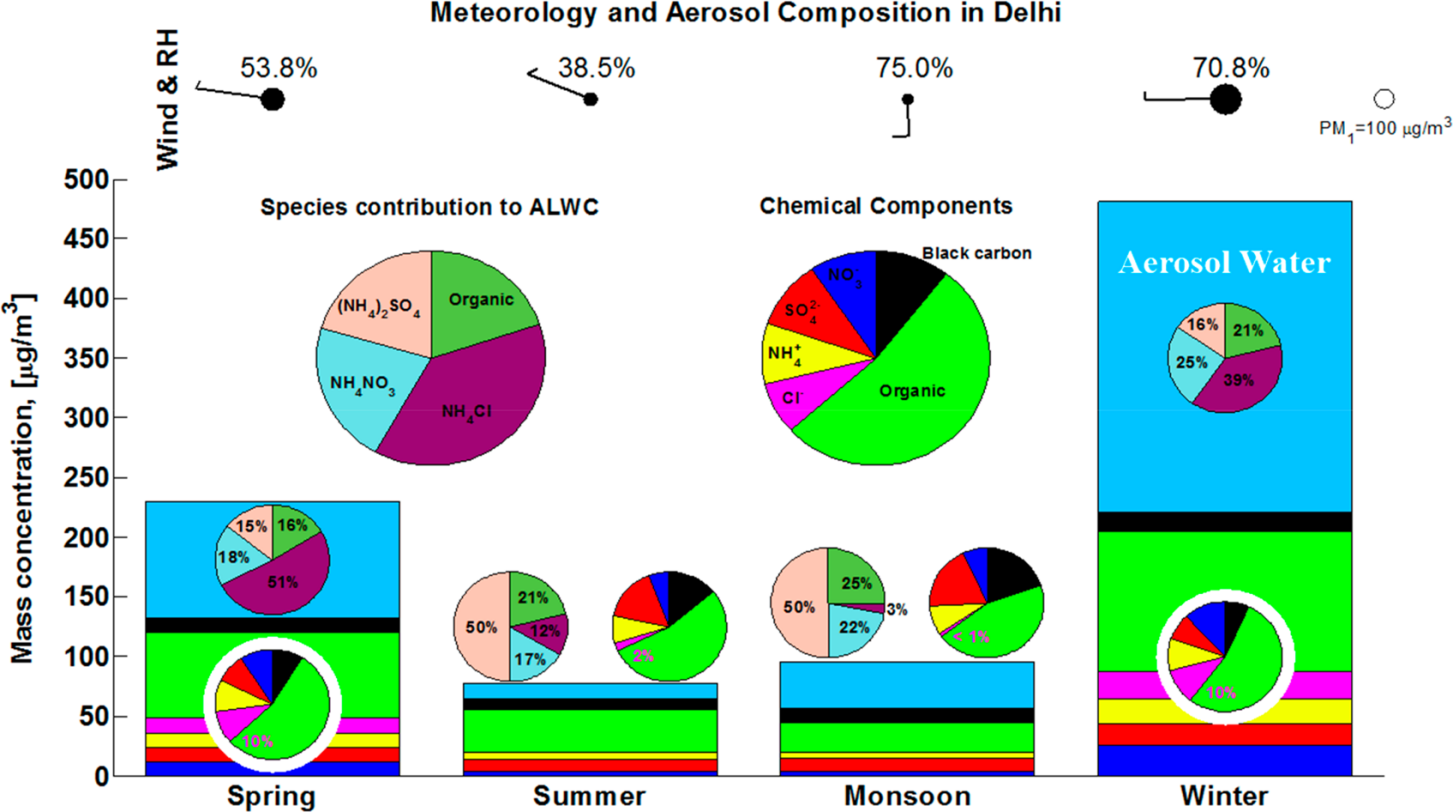
Chemical composition of PM_1_ in Delhi by season. The average PM_1_ mass concentration (size of dot), RH, wind speed, and dominant wind direction are given in the top panel. The relative contributions of each chemical component and the aerosol liquid water content (ALWC) associated with it are given in the pie charts. The large pie charts at the top show the average over the whole period. The pale blue color indicates total ALWC mass concentration.

Figure 1 shows the average mass concentrations of each aerosol component and an estimate of ALWC in each of the four seasons in Delhi; the water-soluble inorganic salts in the liquid aerosol phase estimated by the ISORROPIA model for each season are also given in supplementary Table S1. ALWC is one of the most important components in particulate matter at ambient conditions, contributing about 40% of aerosol total mass in spring and monsoon seasons and about 55% in winter. Winter is the most polluted season, with dry PM_1_ mass of 220 (±87) μg/m^3^, and ALWC contributes an extra 260 (±228) μg/m^3^. This ALWC in Delhi is five times higher than reported during winter in Beijing (45 μg/m^3^).^27^ The maximum daily ALWC in Delhi in January 2017 was 740 μg/m^3^, which is 3.5 times higher than the highest daily value recorded in Beijing (210 μg/m^3^).^26,27^ ALWC in Delhi is greater than at any of the observational sites investigated globally in previous studies.^25,26^ This is consistent with a recent study reporting that aerosol hygroscopicity in Delhi is about twice as high as in Beijing and much higher than in other Asian regions,^19^ despite having very high average organic content.^33^ Although chloride contributes only 10% of dry PM_1_ mass, ammonium chloride contributes ∼40% of water uptake in winter. In contrast, organic components contribute more than 50% of dry PM_1_ mass but are associated with only 21% of ALWC. Spring also shows a high loading of ALWC (98 μg/m^3^ on average) with an average mass-based hygroscopic growth factor of 0.74 (ALWC normalized by dry PM_1_), despite the relatively dry conditions with average ambient RH of 54%. About 10% of dry PM_1_ mass is chloride, and 50% of ALWC is associated with ammonium chloride in spring. The mass loading of surface PM_1_ is lowest in the monsoon season (56 μg/m^3^ on average), partly due to the intensive wet deposition and stronger vertical transport;^28,32,59,60^ but high humidity in this season (RH = 75% on average) can promote the hygroscopic growth of particles, and the average mass-based growth factor is 0.68. In the monsoon season, 75% of ALWC is associated with inorganic components, and only 3% is associated with ammonium chloride due to negligible levels of particulate chloride (<1%). This is probably due to the high solubility of ammonium chloride and hydrogen chloride, which leads to them being effectively washed out. It also suggests that open burning could be an important source of ammonia^61−63^ and chloride in urban regions.^33^ This emission source is suppressed in the monsoon season, because open burning is damped by continuous and intensive rainfall. The PM_1_ concentration is relatively low in summer due to efficient boundary layer mixing,^28^ with an average of 64 μg/m^3^, and summer has the lowest mass-based growth factor of 0.21 due to the relatively dry conditions (RH = 39% on average).

**Figure 2:**
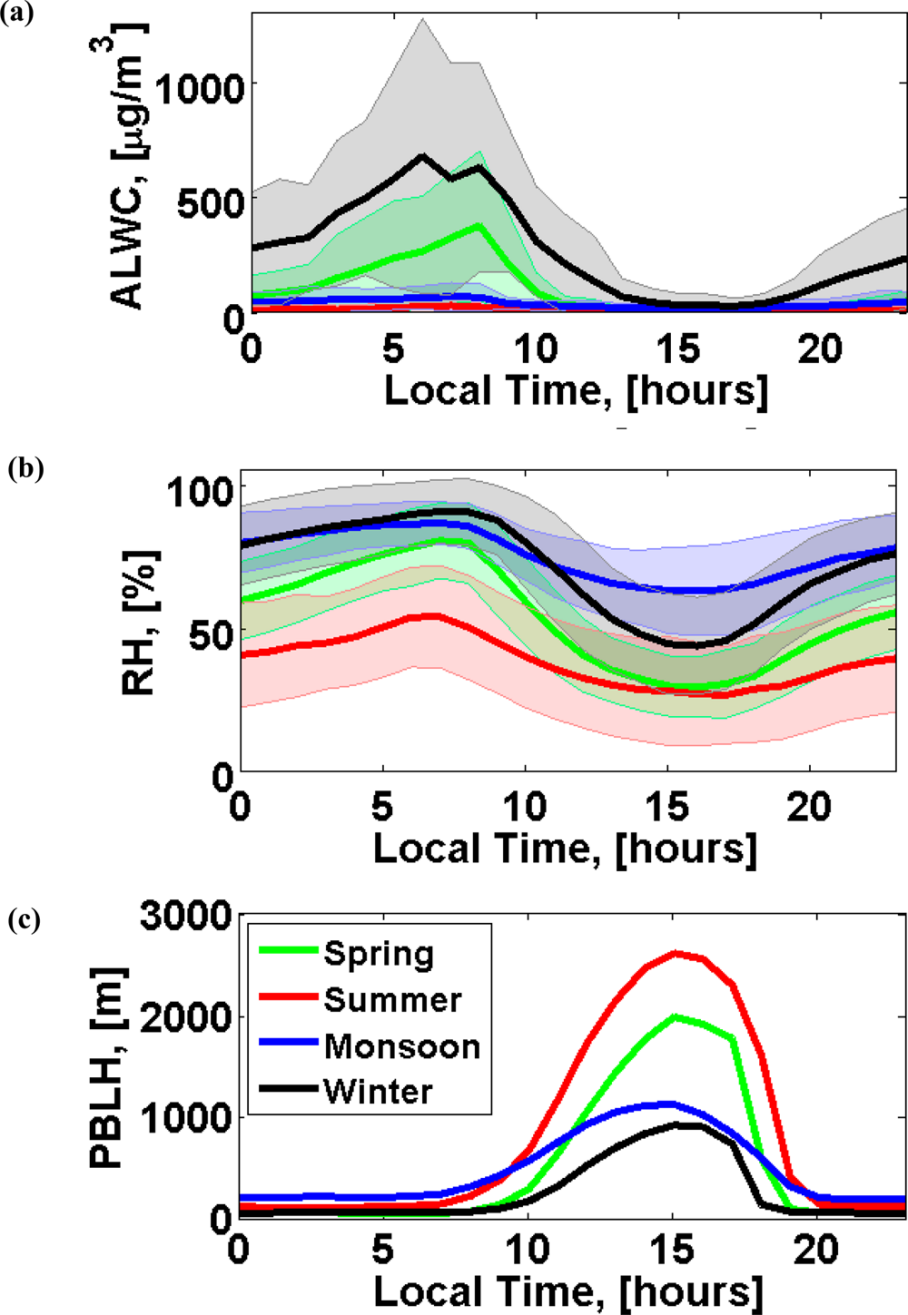
Diurnal patterns of ALWC (a), RH (b), and PBL height (c) in four seasons in Delhi. The shaded areas indicate one standard deviation.

Figure 2 shows the diurnal patterns of ALWC and RH for each season. High concentrations of ALWC are found in winter and spring, with seasonal hourly concentrations peaking at about 630 μg/m^3^ and 380 μg/m^3^ at 6–8 a.m. local time, respectively. Several factors govern high ALWC concentrations in the early morning. The ALWC peaks coincide with the peaks in RH (90% in winter and 80% in spring). The hygroscopicity of particles also peaks around 6–8 a.m., with average κ values (an index of water uptake ability of aerosol) in the range 0.32–0.42^34^ due to the high loading of chloride in the early morning.^33,64^ Furthermore, the shallow PBL in the early morning (Figure 2c) suppresses the dispersal of pollutants and water vapor, leading to increases in PM_1_ and ALWC. ALWC concentrations decrease as the PBL develops after 9 a.m. and approach their lowest values around 4 p.m. when the PBL is fully developed. The development of the PBL dilutes the water vapor and particulate matter and therefore reduces RH, PM_1_, and ALWC concentrations in the afternoon.

### Driving Factors for ALWC in Delhi

**Figure 3:**
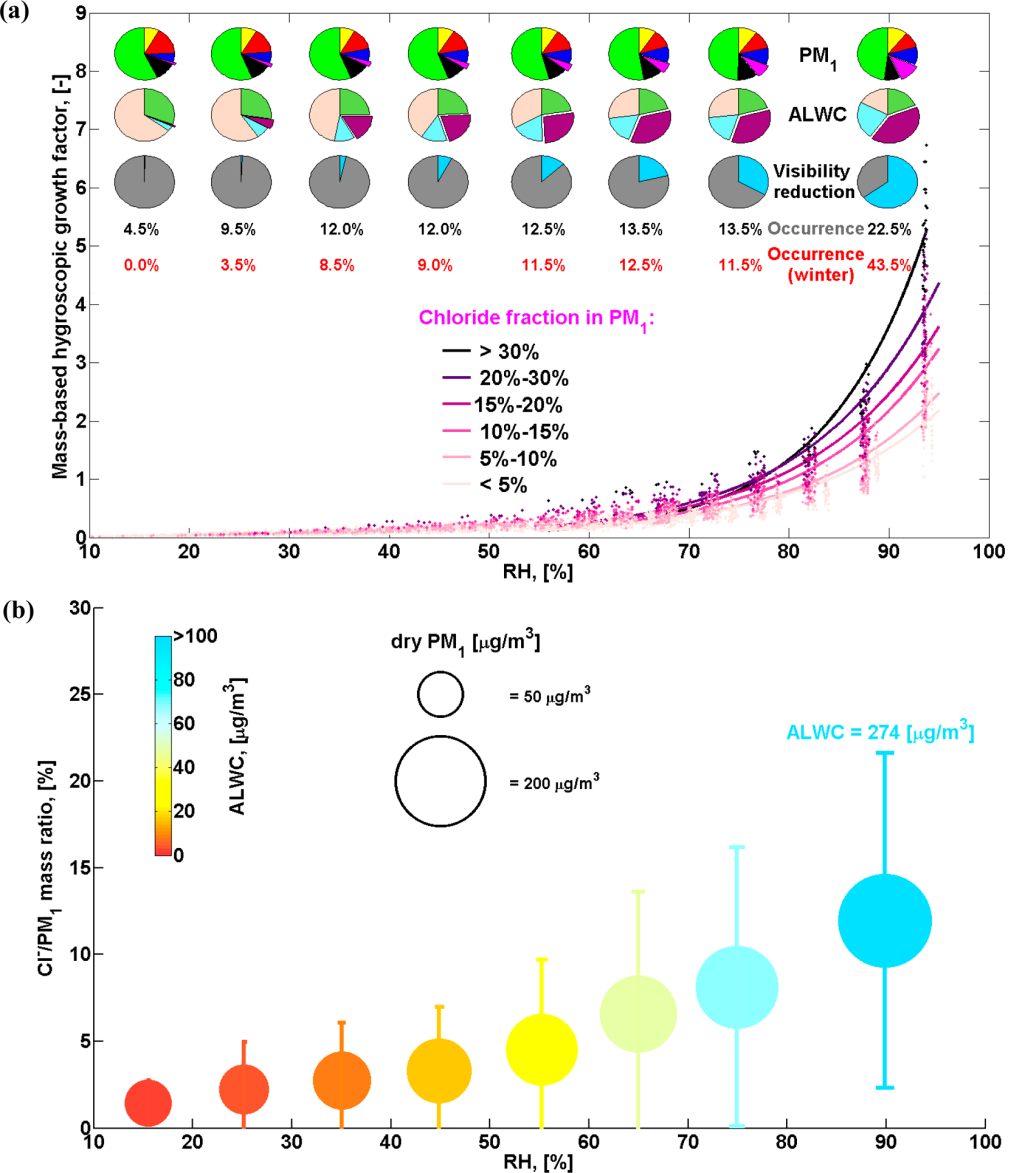
Relationships between ALWC, PM_1_, chloride fraction, and RH. (a) Mass-based hygroscopic growth factor of dry PM_1_ (*y*-axis) as a function of RH for different chloride fractions (indicated by color). The pie charts show the chemical composition of dry PM_1_ (top), the relative contribution of each component to ALWC (middle), and the relative contributions of dry PM_1_ (gray) and aerosol water (pale blue) to visibility impairment (bottom). The pie slices for chloride in PM_1_ and the contribution of ammonium chloride to ALWC are detached. The colors on the pie charts are the same as in Figure 1. The frequency of occurrence of each RH regime is marked in black for the whole period and in red for the winter season. (b) Chloride mass fraction in dry PM_1_ as a function of RH. ALWC is indicated by color, and PM_1_ dry mass concentration is indicated by the size of the circle. The error bars show one standard deviation.

To explore the factors governing ALWC uptake in Delhi, we quantify the ALWC associated with each species using developments in the ISORROPIA model (Figure 3a). The evolution of ALWC with increasing RH and its impact on visibility reduction are also shown in Figure 3. Ammonium chloride is the largest contributor to ALWC, up to 41% when RH > 80%, and ALWC averages 274 μg/m^3^ at this high humidity (pie charts in Figure 3a). Figure 3b also shows an increasing fraction of chloride with an increase in ALWC, and this positive correlation is especially strong at high RH. It is clear that ALWC increases with RH, dry PM_1_, and chloride mass fraction. Larger PM_1_ loadings provide more hygroscopic matter, and higher RH conditions further promote water uptake. On average, ALWC increases from less than 20 μg/m^3^ when RH < 40% and PM_1_ < 100 μg/m^3^ to about 70 μg/m^3^ when RH is ∼75% and PM_1_ is ∼170 μg/m^3^ and then to 274 μg/m^3^ when RH > 80% and PM_1_ > 200 μg/m^3^. The mass fraction of chloride increases exponentially as RH increases from less than 3% for RH < 40% to 12% for RH > 80%.

Similar conditions are seen during the heating season in winter in Beijing, when coal and biomass burning contribute significantly to chloride concentrations and lead to an increase in aerosol hygroscopicity.^44,65^ Ammonium chloride has much higher water uptake potential than other measured components, with a κ of 0.93,^22,51,65,66^ and can co-condense with water vapor to further enhance hygroscopic growth of aerosol particles with increasing RH.^33^ Northwesterly winds are dominant in winter and spring in Delhi when the fraction of chloride is high (10%, Figure 1), suggesting a source of chloride compounds northwest of the city.^32,33^ The high frequency of stagnant weather conditions in winter and spring, indicated by average wind speeds of less than 3 m/s (Figure 1), can inhibit the dispersal of pollutants and also increase ALWC.

To further illustrate the governing role of ammonium chloride in water uptake and the evolution of ALWC with the RH increase, we group the observations according to chloride fraction and investigate the mass-based growth factor as a function of RH (curves in Figure 3a). The observations in each group are fitted with an exponential function of the form *“ALWC/PM*_1_= exp(a * RH + b)”. All groups show good coefficients of determination, with *R*^2^ values ≥0.9. The fitting parameters and *R*^2^ values are given in supplementary Table S2. In general, water uptake increases as ambient RH increases, and the slope becomes steeper with an increasing chloride fraction. This indicates that particles with a larger chloride fraction uptake more water vapor for a given RH increment (Figure 3a). The average chloride fraction also increases as RH increases, and the contribution of ammonium chloride to ALWC increases from less than 5% for RH < 30% to 41% for RH > 80% (pie charts in Figure 3a). One mass unit of dry PM_1_ can uptake 5–7 units of water under conditions with RH > 90% for a chloride fraction larger than 30% but only ∼1 unit of water for a chloride fraction less than 5%. We also observe that the contribution of chloride becomes increasingly important as PM_1_ mass concentration increases (Figure S4) and that severe pollution usually occurs when RH is high (Figure 3). On average, dry PM_1_ increases from ∼50 μg/m^3^ to ∼100 μg/m^3^ and then to 200 μg/m^3^ when RH increases from ∼20% to 40% and then to 80%; the chloride fraction increases from 1.5% to 3% and then to 12%, respectively. Correspondingly, ALWC increases from ∼2 μg/m^3^ to ∼16 μg/m^3^ and then to ∼270 μg/m^3^. It is clear that the increases in ALWC and chloride fraction are enhanced when RH is higher than 60% (Figure 3). This indicates that co-condensation of semivolatile ammonium chloride with water vapor can greatly enhance water uptake and lead to severe haze,^33^ especially under humid conditions (RH > 60%), which occur frequently in Delhi and constitute about ∼50% of the investigation period and ∼70% of winter as a whole (Figure 3a).

### Role of ALWC in Haze Development

Our results show that heavy pollution is typically associated with high ALWC loading, and this may facilitate heterogeneous reactions and condensation of semivolatiles and thus worsen particulate matter pollution. This positive chemical feedback between ALWC and particulate matter formation was recently identified in Beijing in winter.^3^ Key examples of this include nitrate formation via N_2_O_5_ heterogeneous hydrolysis^58,67^ and sulfate formation via SO_2_ oxidation,^54,68^ both of which are demonstrated to be important for pollution in Beijing.^54,69^ Here, we use our comprehensive long-term observations and two detailed case studies to demonstrate the role of ALWC in PM secondary formation in Delhi.

**Figure 4:**
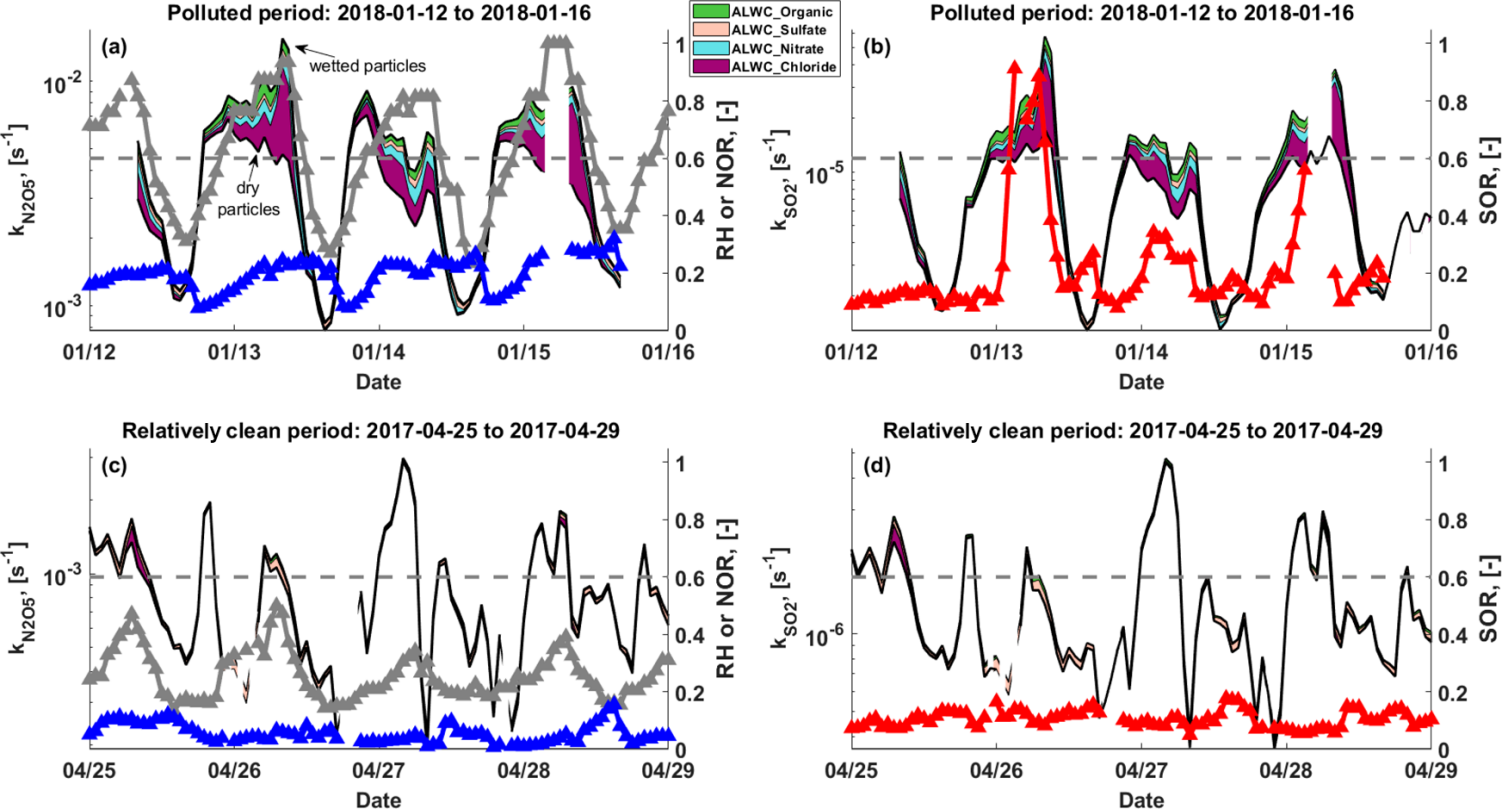
The rate constants (*k*) for heterogeneous loss of N_2_O_5_ and SO_2_. The *k* values for wet and dry particles are indicated by the black lines, with filled colors in between showing the contributions of enhancements from aerosol water associated with different species. Note that the *y*-axis for *k* values is on a logarithmic scale. RH is given by gray lines in panels (a) and (c), with a dashed gray line showing the 60% RH level. Sulfur and nitrogen oxidation ratios are given by red and blue lines, respectively. Two four-day periods are analyzed, with the polluted period shown in the top panels (a, b) and the relatively clean period shown in the bottom panels (c, d).

We first perform a long-term statistical analysis and find that the sulfur and nitrogen oxidation ratios, SOR and NOR, which are often used as an indicator of secondary transformation,^70^ increase with ALWC when ALWC < 350 μg/m^3^ and then saturate when ALWC is higher (Figure S5). Here, SOR = *n*SO_4_/(*n*SO_4_ + *n*SO_2_) and NOR = *n*NO_3_/(*n*NO_3_ + *n*NO_2_), where ‘*n*’ represents molar concentration.^70^ This indicates that ALWC facilitates the secondary formation of sulfate and nitrate from the beginning of haze development, until SOR and NOR approach high levels of ∼0.4 and ∼0.22, respectively. To further illustrate the role of ALWC in secondary formations of nitrate and sulfate via promotion of heterogeneous reactions, we perform two detailed case studies, one in a relatively clean period (average dry PM_1_ = 45 μg/m^3^) and one in a polluted period (average dry PM_1_ = 250 μg/m^3^); see details in the Materials and Methods. These two cases are selected because of the contrast in pollution levels and the availability of the observations needed to estimate heterogeneous reaction rate constants. In the polluted period, Figure 4 shows that secondary formations of nitrate and sulfate are promoted when their heterogeneous formation (*k*_N_2_O_5__ and *k*_SO_2__) increases, and SOR and NOR approach their highest levels. We find that ALWC greatly enhances the heterogeneous formation of nitrate and sulfate via increasing *k*_N_2_O_5__ and *k*_SO_2__ by a factor of 1.6 on average (Figures 4a and 4b). Ammonium chloride associated ALWC contributes ∼60% of this enhancement, and when RH > 60%, the enhancement can approach a factor of 5 which is dominated by chloride associated ALWC. This enhancement effect was not observed in the relatively clean period, when the rate constants for dry and wet particles are very similar (Figures 4c and 4d). Correspondingly, the secondary transformation of nitrate and sulfate is low, with average NOR and SOR less than 0.05 and 0.1, respectively. This evidence clearly demonstrated that ammonium chloride and its associated ALWC play an important role in the development of severe haze events, especially under humid conditions.

In contrast, formation of secondary organic aerosol (SOA) may not be greatly facilitated by ALWC, since the decrease of the organic mass fraction is accelerated as RH and ALWC increase (Figure S6). More detailed analysis is needed to develop deeper insight into SOA formation in Delhi, but we lack the comprehensive observations needed to perform this in the current study. We therefore highlight the value of observations of the properties of volatile organics (e.g., species, volatility, solubility, polarity, etc.) and oxidation radicals (e.g., OH radical) in future studies to help better understand SOA formation and its role in Delhi air pollution.

### ALWC Enhances Visibility Impairment and Suppresses the Boundary Layer

High ALWC can degrade visibility by enhancing light scattering.^19,71^Figure S7a–c shows a strong negative correlation between ALWC and visibility in Delhi. This relationship can be described with a function of the form “y = exp(a * x + b) + c”, with a coefficient of determination (*R*^2^) of 0.54. In general, the visibility is reduced to less than 1 km when ALWC exceeds 500 μg/m^3^. Consideration of ALWC significantly improves the correlation between visibility and particulate matter. The *R*^2^ is only 0.43 when dry PM_1_ is considered alone but increases to 0.57 when ALWC is included. Furthermore, an exponential reduction in visibility is observed with increasing total particulate matter (ALWC + PM_1_) and with increasing ALWC, while a weaker linear reduction is observed with increasing dry PM_1_. Following the approach in refs (19 and 33), we quantify the relative contribution of dry PM_1_ and ALWC to visibility impairment (the bottom pie charts in Figure 3a), using a light extinction enhancement factor due to ALWC, i.e., *f*(RH), which represents the ratio of light extinction coefficient between wetted aerosol and dry aerosol. We find that ALWC contributes to less than 20% of visibility reduction when RH < 60%, but this increases exponentially as RH increases. We estimate that at high humidity (RH > 80%), ∼70% of visibility reduction is attributed to ALWC on average, 40% of which is associated with ammonium chloride. Severe haze usually happens under these humid conditions (RH > 80%, Figure 3b), which occur 22.5% of the time during the 13-month observing period and are dominant (43.5%) throughout the winter in Delhi. These results highlight that ALWC can further degrade visibility frequently and may even dominate visibility impairment during severe haze periods, exacerbating surface and air traffic related economic losses^72^ and increasing traffic accidents.^73^

Light scattering by ALWC can also inhibit the development of the PBL,^74^ because PBL evolution is determined by the surface response to solar heating.^75^ The ALWC is particularly high in the morning (Figure 2a), with *f*(RH) of 2.5 in winter and 1.4 in spring at 10:00 a.m. Using the NCAR-TUV radiation model (see Materials and Methods and Supplementary Section S1 for more details), ALWC is projected to reduce downward surface solar radiation by about 51 W/m^2^ in winter and 23 W/m^2^ in spring at 10 a.m. (near the aerosol optical depth observation time for Terra-MODIS). This reduction in surface solar radiation is estimated to suppress the PBL height by about 27 m (15%) in winter and 16 m (5%) in spring, following the parametrized response of PBL evolution to surface solar heating shown in Figures S7d and S7e. The impact on the PBL is expected to be more profound earlier in the morning when ALWC is higher (Figure 2). Suppression of the PBL reduces the mixing and dispersal of particulate matter and water vapor, increasing the RH and hygroscopic growth of particles, as demonstrated by the inverse relationship between high PBL and high ALWC in Figure S7f. An increase in ALWC further reduces the depth of the PBL and enhances both humidity and pollution. This positive feedback is known to play a key role in the formation of heavy haze in Beijing.^74^ Our study suggests that this feedback could play an even more important role in the development of winter haze in Delhi, given the much higher ALWC and stronger solar radiation. Therefore, chloride and its associated ALWC and their interaction with PBL development are critical for the reported peak in surface pollution in the morning in Delhi.^28^

### Policy Implications

The Indo-Gangetic Plain is one of the most populated regions of the world with high emissions of ammonia and nitrogen- and chlorine-containing gaseous precursors from agriculture, industry, fossil fuel, open burning, and vehicles.^76,77^ Highly hygroscopic particulate constituents, such as ammonium chloride and nitrate, can be formed from these precursors through complex atmospheric chemical processes. Here, we show that aerosol liquid water is extremely high in Delhi and triggers the positive feedback between water uptake and secondary formation and also the positive feedback between water uptake and suppression of boundary layer mixing height. The combination of this feedback driven by aerosol water greatly exacerbates air pollution and degrades visibility. The increase of aerosol water is driven mainly by ammonium chloride under humid conditions. Ammonia originates primarily from agriculture, livestock farming,^10,76^ fossil fuel combustion,^78,79^ and open burning in urban regions.^61−63^ Abatement could be achieved via rationalized fertilization practices, improved animal manure management, reduction in fossil fuel use,^78,79^ and control of burning.^80^ However, an imbalance in the abatement of emissions of ammonia and of chlorine-, nitrogen-, and sulfur-containing precursors poses the risk of acid precipitation.^80,81^ We therefore highlight in particular the regulation of chlorine-containing emissions to improve the air quality in Delhi. This study indicates that aerosol water could be a key factor for haze development in megacities with high fossil fuel, biofuel, and traffic emissions, and the suggested emission intervention strategy may also be effective in other cities across India. This study highlights that future studies providing details of chlorine sources over India are critical to inform policymakers designing and implementing appropriate intervention strategies.

## Supporting Information Available

Section S1, aerosol water inhibits PBL development; Table S1, concentration of secondary water-soluble inorganic aerosol in each season; Table S2, parameters for fitting functions in Figure 3a; Figure S1, cross correlation matrix; Figure S2, ion balance in Delhi; Figure S3, overview of data availability in Delhi; Figure S4, mass fraction of each component in different pollution levels; Figure S5, SOR and NOR as function of ALWC; Figure S6, relationship between ALWC and inorganic and organic aerosols; and Figure S7, relationships between PM_1_, ALWC, and meteorology (PDF)
Output of ISORROPIA model in supplementary data set (ZIP)
