# Supplementary material for: Ammonium Chloride Associated Aerosol Liquid Water Enhances Haze in Delhi, India
Source: Environ Sci Technol. 2022 Apr 28;56(11):7163–73. doi: 10.1021/acs.est.2c00650 (PMC9178790; doi:10.1021/acs.est.2c00650)
Supplement: Supplementary file 1 — es2c00650_si_001.pdf [file es2c00650_si_001.pdf]

Supporting Information

for

Ammonium chloride associated aerosol liquid water enhances haze in Delhi, India

Ying Chen[1,2,3*], Yu Wang[4], Athanasios Nenes[5,6], Oliver Wild[1], Shaojie Song[7,8], Dawei Hu[9], Dantong Liu[10], Jianjun He[11], Lea Hildebrandt Ruiz[12], Joshua S. Apte[13], Sachin S. Gunthe[14,15*], Pengfei Liu[16*]

[1]Lancaster Environment Centre, Lancaster University, Lancaster, LA1 4YQ, UK

[2]College of Engineering, Mathematics and Physical Sciences, University of Exeter, EX4 4QE, UK

[3]Laboratory of Atmospheric Chemistry, Paul Scherrer Institut (PSI), Villigen, 5232, Switzerland

[4]Institute for Atmospheric and Climate Science, ETH Zurich, Zurich, 8006, Switzerland

[5]School of Architecture, Civil & Environmental Engineering, École Polytechnique Fédérale de Lausanne, Lausanne, 1015, Switzerland

[6]Center for the Studies of Air Quality and Climate Change, Institute of Chemical Engineering Sciences, Foundation for Research and Technology Hellas, Patras, 26504, Greece

[7]John A. Paulson School of Engineering and Applied Sciences, Harvard University, Cambridge, MA, 02134, USA

[8]College of Environmental Science and Engineering, Nankai University, Tianjin, 300071, China

[9]Centre for Atmospheric Sciences, Department of Earth, Atmospheric and Environmental Sciences, University of Manchester, Manchester, M13 9PS, UK

[10]Department of Atmospheric Sciences, School of Earth Sciences, Zhejiang University, Hangzhou, Zhejiang, 310058, China

[11]State Key Laboratory of Severe Weather & Key Laboratory of Atmospheric Chemistry of CMA, Chinese Academy of Meteorological Sciences, Beijing, 100081, China

[12]McKetta Department of Chemical Engineering, The University of Texas at Austin, Austin, TX, 78712, USA

[13]Department of Civil and Environmental Engineering, UC Berkeley, CA, 94720, USA

[14]EWRE Division, Dept. of Civil Engineering, Indian Institute of Technology Madras, Chennai, 600036, India

[15]Laboratory for Atmospheric and Climate Sciences, Indian Institute of Technology Madras, Chennai, 600036, India

[16]School of Earth and Atmospheric Sciences, Georgia Institute of Technology, Atlanta, GA, 30318, USA

*Correspondence to: Ying Chen (y.chen6@exeter.ac.uk), Pengfei Liu (pengfei.liu@eas.gatech.edu) and Sachin S. Gunthe (s.gunthe@iitm.ac.in)

Contents of this file

Texts:

Section S1– Aerosol water inhibits PBL development

Tables:

Table S1 – Concentration of secondary inorganic aerosol in each season.
Table S2 – Parameters of fitted functions in Fig. 3a;

Figures:

Figure S1 – Cross correlation matrix between ALWC and different factors;
Figure S2 – Ion balance in Delhi;
Figure S3 – Overview of data availability in Delhi;
Figure S4 – Mass fraction of each component in different pollution levels;
Figure S5 – SOR and NOR as a function of ALWC;
Figure S6 – Relationship between ALWC and inorganic and organic aerosols;
Figure S7 – Relationships between PM_1, ALWC and meteorology.

Section S1: Aerosol water inhibits PBL development

We perform analysis in the morning (7-11 a.m.) in winter and spring to demonstrate that aerosol water can significantly inhibit the development of the PBL in Delhi. These periods are chosen because: 1) concentrations of ALWC and pollutants are high in these seasons with a strong peak in the morning as shown in Fig. 2a; 2) PBL development is mainly driven by solar heating in the morning but could be influenced by additional factors such as surface latent heat in the afternoon[1]; and 3) observations of aerosol optical depth (column-integrated light extinction) are available from Terra-MODIS at around 10:30 a.m. local time. In order to quantify the ALWC-induced reduction in downward surface solar radiation and the corresponding inhibition of PBL development, we perform two steps. First, we use the NCAR-TUV radiation model to calculate the surface solar radiation given the aerosol optical depth from Terra-MODIS, which observes column light extinction due to ambient aerosol including the associated water. We further calculate the surface solar radiation in the absence of aerosol water, assuming that PBL is well mixed and the column f(RH) is the same as that at the surface. This is a reasonable assumption given that most of the particulate matter is constrained within the PBL which is only 200-300 m deep in winter and spring at the Terra-MODIS observation time (see Fig. 2c). The ALWC-induced reduction in surface solar radiation can be estimated from the difference between the calculations. The total column ozone loading, aerosol single scattering albedo and surface albedo are considered in the TUV calculations, as detailed in the Methods section. Secondly, we derive a function to describe the relationship between downward surface solar radiation and PBL height in winter and spring, using ERA5 reanalysis. A good relationship ($R^2 > 0.7$) is found as shown in Fig. S7d and S7e. This relationship is used with the ALWC-induced solar radiation reduction derived above, to quantify the suppression of PBL height. It is worth noting that the ERA5 reanalysis does not consider non-sea-salt chloride and its associated water, and therefore the influence of ALWC on surface solar radiation and PBL height are not fully reflected in the ERA5 dataset. Nevertheless, it still represents the evolution of the PBL under surface heating from solar radiation, which is influenced by the climatological and surface physical characters[1]. The influence of ALWC on surface solar radiation is assessed in the first step and the PBL suppression is derived in the second step.

Table S1. Concentration of secondary water-soluble inorganic aerosol in each season. The seasonal average mass concentration of each species in the liquid aerosol phase are estimated using the ISORROPIA model. Note that these species are present as ions in the liquid phase.

Units: μg/m^3	**Spring**	**Summer**	**Monsoon**	**Winter**
Ammonium sulphate	13.9	12.7	12.2	17.8
Ammonium nitrate	12.6	3.0	4.1	23.9
Ammonium chloride	18.4	1.9	0.6	27.0

Table S2. Parameters of fitted functions, in the form *"y = exp(a * x + b)"*, between RH and ALWC with respect to PM_1 dry mass concentrations. The fitted curves are shown in Fig. 3a.

**Data Groups**	**a**	**b**	**Data Number**	**R^2**
Cl fraction > 30%	0.099	-7.660	69	0.93
20% < Cl fraction ≤ 30%	0.080	-6.150	171	0.92
15% < Cl fraction ≤ 20%	0.077	-6.007	202	0.92
10% < Cl fraction ≤ 15%	0.079	-6.351	323	0.90
5% < Cl fraction ≤ 10%	0.076	-6.284	872	0.91
Cl fraction ≤ 5%	0.071	-6.000	2586	0.91

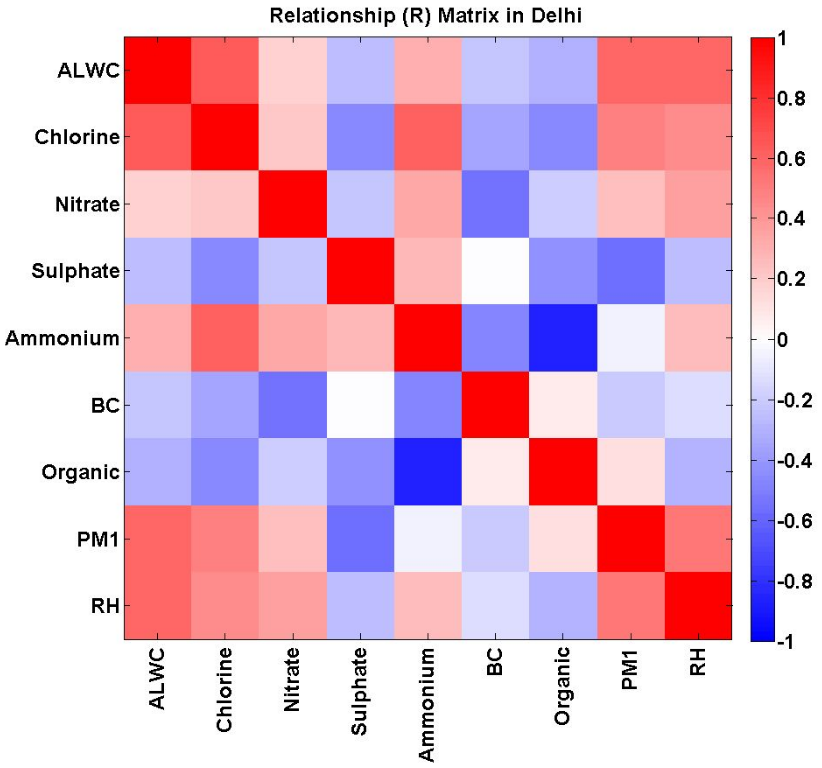

Figure S1. Cross correlation matrix. The colour indicates the correlation coefficient (R) between each pair. ALWC and PM_1 represent the mass concentrations of aerosol liquid water content and particulate matter with diameter less than 1 μm, and RH is the relative humidity. The mass fractions in PM_1 of chlorine, nitrate, sulphate, ammonium, black carbon (BC) and organic are also shown.

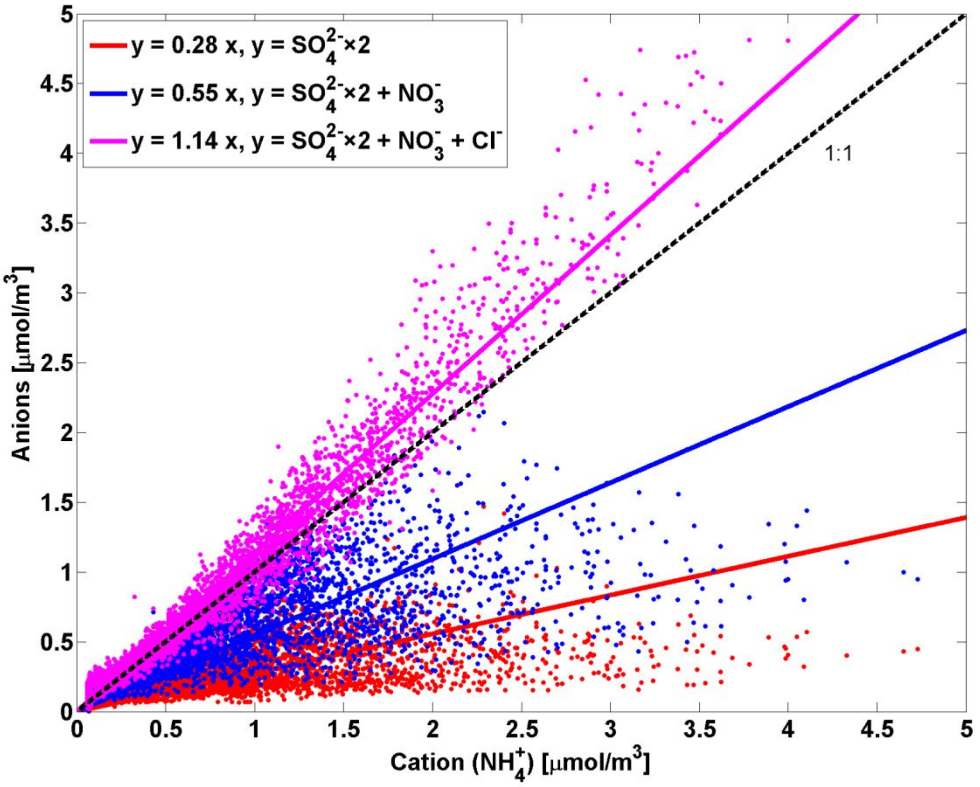

Figure S2. Ion balance in Delhi measured by the ACSM. The colored lines indicate anion-to-cation charge ratios for sulphate (red), sulphate + nitrate (blue), and sulphate + nitrate + chloride (purple), and the black dashed line denotes a 1:1 ratio.

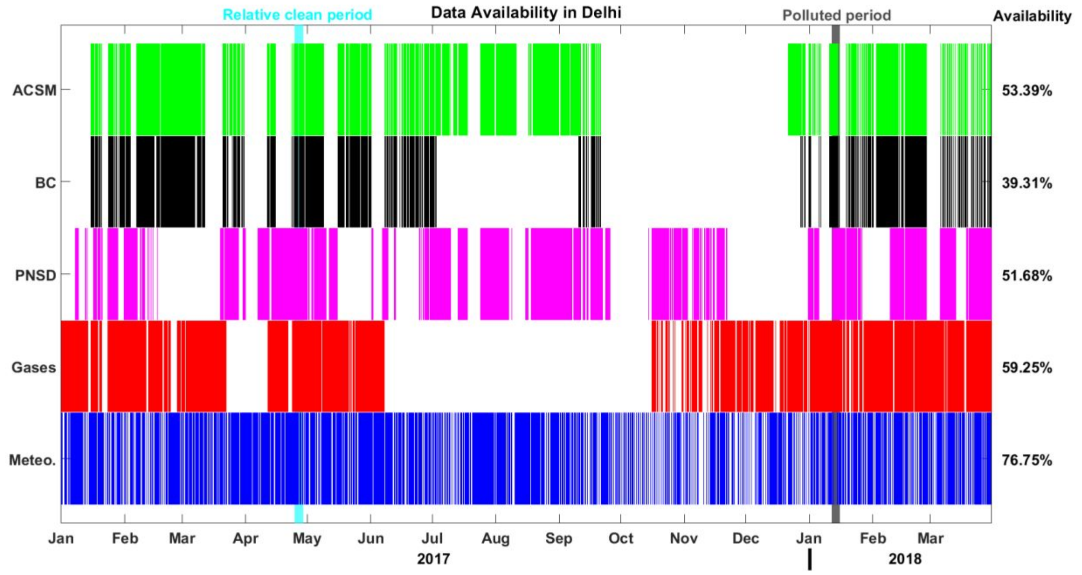

Figure S3. Data availability of hourly observations in Delhi. Non-refractory chemical composition observed by ACSM as shown in green, black carbon (BC) observed by AE33 in black, particle number size distribution (PNSD) in magenta, SO_2 and NO_2 concentrations in red, and meteorology in blue. The overall availability during the whole period, 2017-01-01 to 2018-03-31, is shown on the right side. The relatively clean period (light blue) and polluted period (grey) selected for analysis in Fig. 4 are also marked.

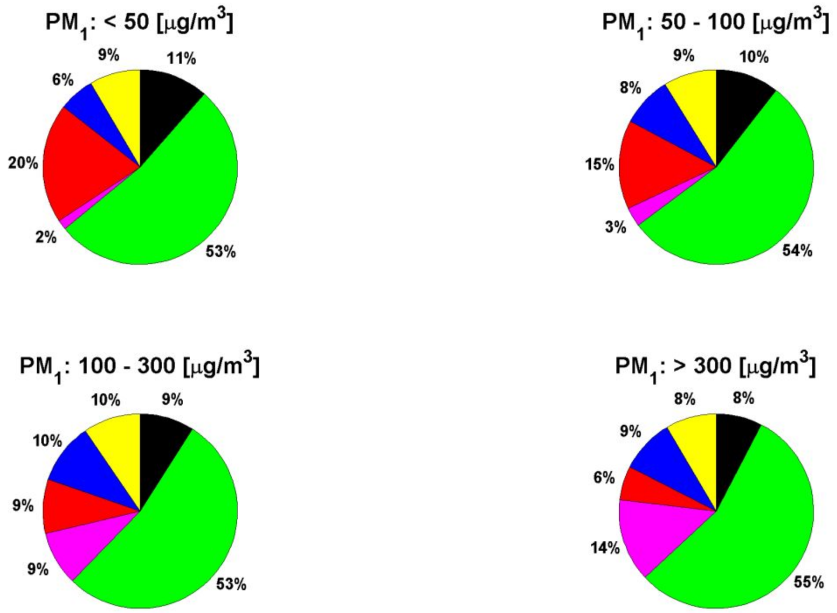

Figure S4. The mass fraction of each component in PM$_1$ at different pollution levels. Ammonium (yellow), nitrate (blue), sulphate (red), chlorine (magenta), organics (green) and black carbon (black).

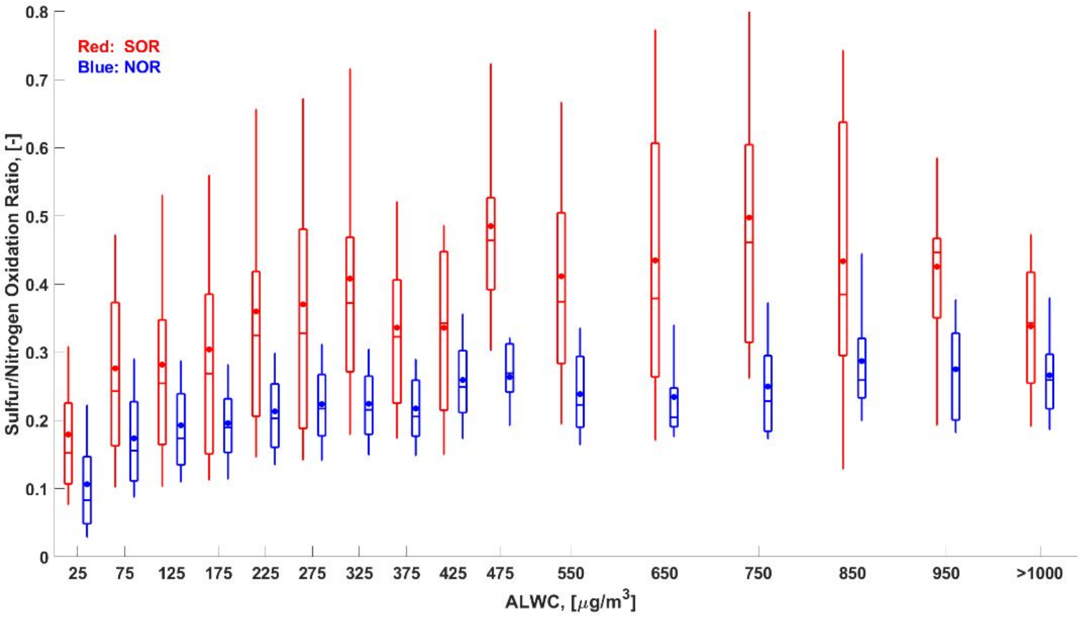

Figure S5. Relationship between aerosol liquid water content and sulfur or nitrogen oxidation ratio, i.e., SOR (red) or NOR (blue). The boxplots show 10th, 25th, median,75th and 90th percentiles with the mean value indicated by a dot.

(b)

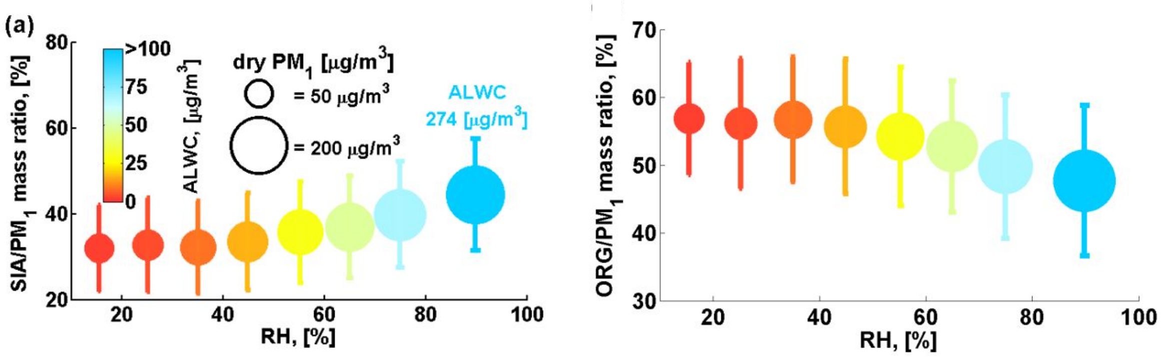

Figure S6. Similar to chloride in Fig. 3b, but showing the relationships between ALWC, RH and the mass fractions of total secondary inorganic (a) and organic (b) aerosols.

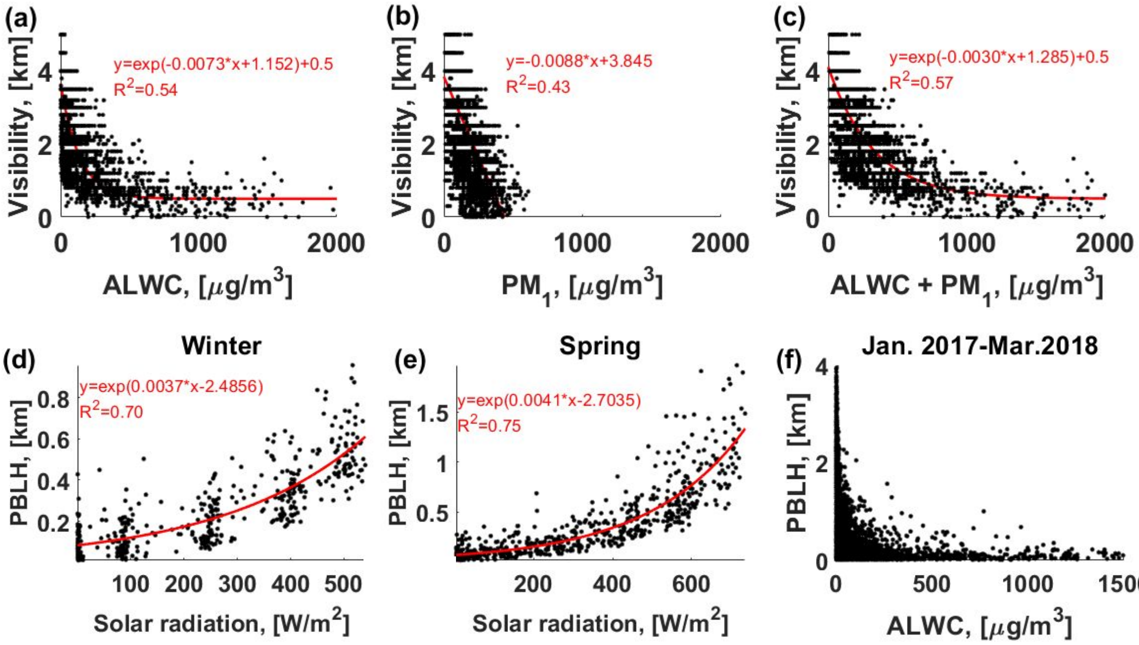

Figure S7. Relationship between PM_1, ALWC and meteorology. Visibility as a function of the mass concentration of ALWC (a), PM_1 dry mass (b) and the sum of ALWC and dry PM_1 (c). Planetary boundary layer height (PBLH) as a function of downward surface solar radiation during 7-11 a.m. in winter (d) and in spring (e), and as a function of ALWC (f).

References:

1. Medeiros, B.; Hall, A.; Stevens, B., What Controls the Mean Depth of the PBL? Journal of Climate 2005, 18, (16), 3157-3172.
